# Co-Creating a District-Wide Professional Development Program and Implementation Model for Trauma-Informed Schools

**DOI:** 10.3390/bs15060726

**Published:** 2025-05-24

**Authors:** Megan Blanton, Erum Nadeem, Pamela Vona, Anusha Sahay, Olivia Kycia, Chris Dudek, Jade Garcia, Candace Coccaro

**Affiliations:** 1 Graduate School of Applied and Professional Psychology, Rutgers University, New Brunswick, NJ 08854, USA; erum.nadeem@rutgers.edu (E.N.); as5266@gsapp.rutgers.edu (A.S.); olk3@gsapp.rutgers.edu (O.K.); cdudek@gsapp.rutgers.edu (C.D.); 2 Center for Safe and Resilient Schools and Workplaces, Los Angeles, CA 90012, USA; pamela@safeandresilient.org; 3 Department of Student Life and Services, Jersey City Public Schools, Jersey City, NJ 07305, USA; jgarcia3@jcboe.org (J.G.); ccoccaro@jcboe.org (C.C.)

**Keywords:** trauma-informed schools, research practice partnership, mixed-methods, multi-tiered systems of support

## Abstract

Research practice partnerships (RPP) between schools and researchers present a promising approach to co-creating scalable professional development for trauma-informed schools. This study used an RPP to develop an implementation model for a trauma-informed professional development program across 15 schools in a major urban school district. The primary study goal was to describe the RPP’s co-design processes used to develop and mount a large-scale professional development program with accompanying implementation supports. A secondary goal was to provide representative case examples of feedback loops for real-time improvements to the implementation strategies. A rapid mixed methods approach drawing on the principles of developmental evaluation was used to collect implementation process data including RPP team meeting notes and documents, informal discussions, training and survey completion reports, attendance, and implementation workshop exit tickets. These data were triangulated to conduct preliminary analyses which were then presented to RPP team members for collaborative review. Results highlighted seven co-designed elements of the TISE implementation support system—engaging and supporting school leadership, implementation teams, live and asynchronous training, ongoing consultation, delivering practical resources, relationship building, and continuous improvement. Exemplar feedback loops highlighted immediate improvements to implementation resources via exit tickets and enhanced strategies for building long-term school-level team effectiveness and engagement via attendance tracking.

## 1. Introduction

Children in the United States are exposed to alarming rates of potentially traumatic experiences (PTEs) with approximately 64% of people experiencing at least one exposure before age 18 and 14% of children facing four or more exposures (Swedo et al., 2023). Exposure to 4 or more PTEs before the age of 18 is significantly associated with poor physical and mental health, including increased frequency and quantity of alcohol use, trauma-related distress, depressive symptoms, anxiety symptoms (C. Overstreet et al., 2017), and lower academic achievement including poor school attendance, behavior issues, and failure to meet grade-level standards (Blodgett & Lanigan, 2018). However, recent research has demonstrated that a variety of protective factors may buffer the impact of PTE exposure including social support, school connectedness, and close family relationships (Ozer et al., 2015; Schwerdtfeger Gallus et al., 2015). Further, a variety of promotive factors including internal assets such as coping and racial identity, and resources such as mentoring relationships, safe school and home environments, and positive childhood experiences have been demonstrated to promote resilience (Han et al., 2023; Terrana & Al-Delaimy, 2023; Zimmerman et al., 2013).

Schools are the ideal context to buffer the impact of PTE exposure among youth as their structure, policies, and practices lend themselves to developing these protective and promotive factors in children’s lives. Schools that are aware of childhood trauma and make efforts to buffer children from the impacts of trauma exposure are called trauma-informed or trauma-sensitive schools (Wiest-Stevenson & Lee, 2016). Specifically, these schools realize the prevalence of childhood trauma, recognize the symptoms of PTE exposure, respond appropriately to traumatized children, and actively work to prevent re-traumatization within the school environment.

However, many teachers do not receive training in recognizing and responding to childhood trauma (Garay et al., 2022) and feel unprepared to address the consequences of childhood trauma in their classrooms (Alistic, 2012). Thus, many school districts have begun to offer trauma-informed tier 1 training and professional development to their staff (S. Overstreet & Chafouleas, 2016) as part of multi-tiered systems of support (MTSS) for social–emotional and behavioral supports. MTSS is a framework increasingly used by school districts to address students’ academic, behavioral, social–emotional needs. The framework uses a public health approach with tiers of prevention and intervention to support the needs of the entire school population (Nadeem & Forman, 2023). There is growing recognition that implementing trauma-informed approaches within an MTSS framework is likely to contribute to improved implementation and sustainability (Chafouleas et al., 2016; Maynard et al., 2019). Current tier 1 trauma-informed training efforts in schools are often characterized by isolated workshops provided to teachers (Goldenthal et al., 2024; McIntyre et al., 2019). These workshops are typically didactic in nature and provide teachers with information about the prevalence, nature, and consequences of childhood exposure to PTEs. While isolated workshops are useful in increasing teacher knowledge, without a consistent, system-level effort, the school is unlikely to become trauma-informed in a way that integrates knowledge into sustainable policies and day-to-day practices. For example, previous research has demonstrated that there is little skill transfer to the classroom following one-time professional development workshops (Joyce & Showers, 2002). Thus, schools that are aiming to become trauma-informed and engage in trauma-informed practices would benefit from system-wide professional development that includes a form of consistent follow-up.

Some important efforts have been made in schools to create more comprehensive trauma-informed professional development programs that include ongoing consultation and follow-up for teachers or multilevel systems of supports (Dorado et al., 2016; Holmes et al., 2015; McConnico et al., 2016; Pfenninger Saint Gilles, 2016). These efforts have yielded promising outcomes both at the educator and student level including increased self-efficacy for educators (McConnico et al., 2016), increased educator-reported on-task student behavior, decreased suspensions (Dorado et al., 2016), and decreased student internalizing symptoms (Pfenninger Saint Gilles, 2016). However, participating schools were often selected because they had demonstrated the capacity to sustain trauma-informed efforts (e.g., Dorado et al., 2016). Thus, our ability to generalize these results to everyday schools with fewer resources, and our understanding of the extent to which these trauma-informed programs can be scaled, is limited. Therefore, there is a need to study how to tailor professional development programs and implementation supports so that trauma-informed practices are sustainable and scalable across schools with a variety of resource levels.

Despite the limitations in the field, existing trauma-informed school training and implementation efforts have promise and are being adopted widely. Such efforts also align and integrate well with social–emotional learning programs, culturally responsive teaching, and equity-focused, restorative practices (Osher et al., 2021). Moreover, attention from policymakers and funders on trauma-informed systems has galvanized schools and districts across the nation to launch professional development and systems change efforts. In embarking on such efforts, schools have many considerations including the scale at which to implement, alignment with resources and other initiatives, the fit and ease of programs, and multi-stakeholder buy-in. Based on these considerations, districts may decide to engage in pilot work, or they may decide to scale-up immediately based on the existence of social capital, political will, and a desire to ensure equitable access to training and support for all schools. For districts balancing these issues, research-practice partnerships (RPPs) can be particularly helpful RPPS are increasingly recognized as a way to improve implementation of evidence-based, practice-oriented solutions to problems in varied settings (Coburn & Penuel, 2016). Defined by a mutualistic, long-term commitment to addressing shared goals, RPPs bring together researchers, practitioners, and local leaders to identify problems and develop multi-system, sustainable solutions. Recent work has illustrated their positive impacts, both on members and populations served (Penuel et al., 2020). The current study is focused on a school district-university RPP focused specifically on the development of professional development training and implementation model for trauma-informed schools.

Much of the extant literature on professional development, training, and implementation related to social–emotional support, behavior, and school mental health broadly is contextualized within investigator-led randomized control trial and observational research studies (Lyon et al., 2024). The context for the current study is unique because the scope and scale of the professional development effort were determined by school district leadership. Research partners were engaged to support evaluation, the development of a theoretically grounded and empirically driven approach to training and implementation, and to support real-time changes based on immediate feedback and continuous improvement. The partners in the current study include a large urban school district and a large public university. As one component of a broader effort to launch trauma-informed MTSS, the district leadership decided to engage school principals, leadership teams, and all school staff in a program called Trauma-Informed Schools for Educators (TISE; Wong et al., 2024), a professional development program that equips educators to provide Tier 1, universal, trauma-informed supports to their students. With supportive infrastructure from federal grant funding, the district’s goal for TISE implementation is to train all schools’ staff (instructional and non-instructional) across 39 schools in TISE and to engage in three core practices from the program that were aligned with district school climate improvement efforts (i.e., community-building circles, warm and intentional greetings, and mindfulness activities). Other components of the RPP’s efforts include building and implementing Tier 2 and Tier 3 classroom level and mental health supports for students (e.g., training clinicians in evidence-based treatments, parent support programs).

As noted above, the current study focuses on elucidating the collaborative processes used for the co-creation of the TISE professional development program across a first cohort of 15 schools in the first year of TISE implementation. Evaluation of program outcomes (e.g., training uptake, practice use) from this multi-year effort will be reported in future studies. The RPP’s implementation approach was informed by research on the identification of effective and commonly used implementation strategies (Powell et al., 2011) and conceptual models of multi-level implementation drivers (Damschroder et al., 2009; Fixsen & Blasé, 2008; Fixsen et al., 2013). User-centered design approaches were deployed to tailor implementation strategies for taking TISE to scale using existing resources in the local context (e.g., Dopp et al., 2019, 2020; Haines et al., 2021).

Using implementation process and continuous improvement data from the first year of implementation, the current study seeks to build the literature on RPP processes with schools and elucidate real-world implementation approaches and improvement processes that could be used by schools and districts. This study has two specific goals. The first goal is to describe the RPP processes used to co-design and mount a large-scale professional development program with attention to theoretical drivers of implementation and scalable structures and supports. The second goal is to provide specific case examples of data-driven process improvement efforts that were used to make real-time improvements to the implementation support structure.

## 2. Materials and Methods

### 2.1. Partnership Approach

Broadly, our RPP is guided by Wells et al. (2006) model for community-academic partnerships which begins with the negotiation of priorities across partners and emphasizes evidence-based practice, evaluation, implementation, and sustainability built on local strengths and resources. In the current partnership, there is a core team at each of the three institutions. At the school district, core team members include the department director and cross-disciplinary project leaders (social work, school psychology, teaching), with consultation from frontline team members and senior district leaders such as the superintendent, school board, and parent advisory councils. At the university, the project team consists of evaluation and implementation support staff including faculty, project directors, clinical supervisor, postdoctoral researcher, and research assistants. Finally, the TISE purveyor organization was represented by the TISE curriculum developers and support staff.

### 2.2. History and Collaborative Structures

The school district enrolls 26,782 students ages 3–21 (38% Latinx, 27% Black, 18% Asian, 14% White, 3% others); 78% qualify for free or reduced lunch, 14% are classified with disabilities, 13% are English Language Learners. Forty-eight percent of students identify as female and 52% as male. Prior to the district’s current efforts to develop trauma-informed MTSS, the university and district partners had collaborated on several smaller research and practice projects (e.g., pilot studies, needs assessments, professional development efforts). This foundational work led to the collaborative submission of grants and the acquisition of federal funding related to developing trauma-informed MTSS by the district. The goals of this grant include (1) developing an MTSS operational framework, (2) providing comprehensive training and implementation support for educators in trauma-informed classrooms and student-targeted trauma-informed approaches, (3) developing trauma-informed mental health screening, referral, and treatment services, (4) increasing awareness of the impact of trauma among families and community members, and (5) developing a sustainability plan for these supports. The TISE professional development program was developed to meet the second goal of the grant.

To run the grant, several collaborative structures were established. Core weekly research-practice meetings included: (1) research and evaluation, (2), program and implementation planning, (3) practice issues, and (4) TISE implementation. Additional team meetings were based on the need for group working time, deliverables, or were project- or initiative-specific, and included additional partners (e.g., partnering clinics, community organizations, advisory groups). Finally, a training-of-the-trainer (ToT) mechanism was established to train current district staff to deliver the TISE live training. This mechanism is crucial to sustain the district’s trauma-informed MTSS.

For the TISE program implementation component of this collaboration, the RPP members worked together to develop program goals. Core benchmarks for success included: (1) successfully building and launching a multilevel and multi-component implementation support structure for trauma-informed schools, (2) building infrastructure for data-based decision making and continuous improvement of the implementation support structure (3) engagement in the implementation process by school staff (i.e., participation, engagement, and perceived benefit of coaching), (4) uptake of the asynchronous TISE training by school staff, and (5) use of trauma-informed practices by school staff. The current paper focuses on the first three indicators of success by focusing on implementation structures (study goal 1) and improvement processes (study goal 2). Though outside of the scope of the current study, examination of indicators four and five is also underway.

### 2.3. Implementation and Co-Design Process

Using frameworks and practices from user-centered design, the RPP team leveraged joint expertise to co-create implementation strategies within our team meetings. For the current study, we focus on the work that occurred in the TISE-specific meetings. User-centered design is an iterative approach to increasing the usability of innovations by obtaining input from key stakeholders and improving the relationship between the practice and the context. It is typically organized into a discover-design-build-test framework in which end users provide critical input into each aspect of program or intervention development and improvements are made in iterative cycles (Lyon et al., 2019). Applied to implementation, the specific foci are integrating the new practice with the context and selecting well-fitting implementation strategies. The process involves selecting and tailoring implementation strategies based on context, workflows, and resources using co-creation methods (e.g., Haines et al., 2021).

In our study, this involved weekly co-design meetings in which all partners contributed their experience, knowledge, and expertise across research and practice. For example, the research team provided expertise in implementation strategies that focused on implementation drivers related to competency, organizational support, leadership support, and top-down and bottom-up feedback loops (e.g., Fixsen & Blasé, 2008; Fixsen et al., 2013; Powell et al., 2011). Competency drivers are strategies to implement an intervention as intended (e.g., coaching, training). Organizational-level drivers address the environments needed for the delivery of effective services and programs (e.g., buy-in, collaboration, implementation teams). Leadership drivers are focused on leadership support and strategies leaders can use to support change in practice (Fixsen & Blasé, 2008). Implementation models also emphasize top-down and bottom-up feedback loops where information and learning is bidirectional between leaders and frontline staff (Fixsen et al., 2013). Based on their knowledge of implementation theory and strategies, collaborative learning models, continuous improvement, and coaching and consultation, the researchers supported the initial strategy selection.

The TISE purveyor organization provided expertise in the TISE training and practice elements, and implementation experience drawn from a range of sites across the nation. The district team provided context-specific expertise that allowed tailoring of the strategies. Specifically, they had critical expertise about organizational structures and norms, organizational climate and culture, communication channels, workflow, resources and time constraints, past implementation successes and failures, educational practice improvement, and need, fitness, and relevance. All decisions were documented in weekly meeting notes, workflow documents, schedules, and co-working products. All materials were stored in shared drives. The current study focuses on the implementation of trauma-informed skills for educators training and implementation for a first cohort of 15 schools in the district. This included five elementary schools, five pre-kindergarten through 8th grade schools, two middle schools, and three high schools. Schools were chosen by the director of the department in conjunction with the superintendent based on their history working with each school and their perception of the schools’ readiness for trauma-informed skills and fitness with the schools’ needs. In addition, they wanted to ensure that they had representation across elementary, middle, and high school.

### 2.4. TISE Curriculum

Trauma-informed Skills for Educators (TISE) is a curriculum designed to increase educators’ knowledge about trauma and equip educators with skills and strategies they can use to create a safe and supportive environment for youth exposed to trauma. This curriculum was selected to serve as the foundational content of the districtwide trauma-informed professional development effort. The curriculum is grounded in SAMHSA’s four Rs and 6 principles (SAMHSA, 2014). The first four modules: (1) What is Trauma, (2) Neurological Impact of Trauma; (3) Symptoms of Trauma; (4) Understanding Resilience, aim to equip educators with a trauma lens helping them to better assess how trauma may be contributing to student well-being, behavior and performance. The remaining modules (5) Classroom Environment; (6) Trauma-informed Communication to Build Supportive Relationships; (7) Trauma-informed De-escalation; (8) and Trauma-informed Staff Collaboration are designed to equip educators with skills they can use every day to establish a safe and calm classroom environment, build supportive relationships with students, de-escalate disruptive behaviors, and promote a trauma-informed school climate which includes educator peer support.

Currently, there are three modalities through which the TISE curriculum can be delivered. The first is an online asynchronous course. The course contains eight modules and is about two hours in length with each module taking about 15–20 min to complete. Each module includes vignettes and other interactive exercises to aid in knowledge acquisition and to help educators apply the knowledge to their own past and current experiences with students. Modules 6 and 7 include exercises that give users the opportunity to practice communication and de-escalation skills within a gamified simulation. The TISE curriculum can also be delivered as a six-hour professional development. This model allows for a deeper discussion of the core concepts, opportunity to practice skills, and implementation planning. Finally, TISE can be delivered through a more intensive coaching curriculum. This can be achieved through monthly virtual sessions that cover a particular TISE concept or skill each month

These varied training modalities provide options for creating a tailored and systematic training approach that allows for both broad reach and depth of knowledge. The online asynchronous is advantageous in its ability to reach all educators by removing common logistical barriers (e.g., district professional development times, calendar and time constraints). Live training allows for a more intensive training experience that includes reflection, discussion and the opportunity to practice skills and receive feedback.

Finally, the TISE Curriculum has an accompanying coaching and consultation series. This includes up to nine 90 min sessions that dive deeper into trauma-informed practices and allow for cultural adaptations and tailoring content to the local context. In the current project, these sessions were conducted with school climate leadership teams (SCLTs) from each school. While the specific composition of each SCLT varied by school site, they typically included an administrator, student support staff (school counselors), and classroom teachers. The primary responsibility of the SCLT was to work on school climate in line with state initiatives and foster a school environment conducive to academic success. Because of this focus, they were seen as the ideal team to participate in the ongoing implementation support.

### 2.5. Procedures

Our overall methodological approach is grounded in principles of developmental evaluation (Gamble, 2008; Patton, 2011). In contrast to traditional evaluation focused on program outcomes and effects, developmental evaluation is primarily focused using evaluation methods to improve a project or process in real time. It is designed to examine dynamic processes as they emerge to improve and support innovation. There is a recognition that nonlinear processes, uncertainty, and ambiguity are part of the process in complex systems (Patton, 2011). Data collection using this framework can include traditional (e.g., survey data) and nontraditional methods (e.g., case studies, experiential data-gathering, interviews, observations of naturally occurring decisions and processes; Gamble, 2008; Patton, 2011). These procedures are congruent with rapid assessment procedures that have been used in the context of implementation research focused on implementation context, process, barriers, facilitators and outcomes, including participant observation and mixed methods approaches (Hamilton & Finley, 2019; Palinkas & Zatzick, 2019).

For the current study, which was focused on delineating the implementation support structure and improvement processes, the RPP planned a priori in which data sources would be routinely gathered and tracked. Specifically, we used team notes from coaching, meetings, and site visits, team agendas, training materials (e.g., PowerPoint slides, handouts) and coaching agendas for the qualitative review of the implementation support structure. The training surveys and completion reports were used as a core element of the continuous improvement structure. The remaining data sources were used to illustrate case examples for continuous improvement and process improvement (e.g., TISE-related completion reports, attendance by SCLT members at training and coaching sessions, and immediate feedback from coaching sessions). All procedures were reviewed by the university IRB.

### 2.6. Data Sources

#### 2.6.1. Archival RPP Materials

In line with developmental evaluation approaches (Gamble, 2008; Patton, 2011) and rapid qualitative methods in implementation science (Palinkas & Zatzick, 2019), information-gathering occurred within the context of ongoing work on collaborative projects. This included meeting agendas, team member notes, shared products and working documents, training materials, informal discussions, and e-mail exchanges among the team members.

#### 2.6.2. Implementation Process Data

TISE completion reports and survey. Asynchronous TISE training progress was monitored by university-based evaluators in collaboration with the TISE purveyor team. Before and after completing TISE, school staff were asked to take part in a survey regarding their attitudes, beliefs, and feelings about different aspects of their jobs and personal wellbeing. Although survey data are not reported in the current study, it is a data source that will be used by the district as another indicator of progress and long-term change.

Completion of this survey was required before course access was provided. Data on completion of the survey and course was regularly monitored to indicate staff that had “not started”, “incomplete” or “complete” progress.

Attendance at educator implementation workshops. Attendance by SCLT members at virtual implementation workshops was tracked with a brief Google Form at the beginning of each session. All attendees were asked to complete the form to receive credit for their participation that day. The form asked participants for their names, email addresses and the name of the school at which they work.

Exit ticket from implementation workshops. Exit tickets were brief, electronic surveys developed by the RPP research team in collaboration with school district personnel administered via Qualtrics at the conclusion of each implementation workshop. Exit tickets were designed to contribute to continuous improvement efforts and facilitate developmental evaluation. Accordingly, participants were asked to provide feedback on their progress with TISE implementation and the quality of implementation workshops. Participants were asked to report at least one trauma-informed skill learned from the previous implementation workshop that they had used in the past month. To obtain feedback on the utility of each workshop, participants were asked two questions about the implementation workshop they had just completed: how helpful the workshop was on a scale of 1–100 using a visual slider bar format, with 1 being the least helpful and 100 being the most helpful, and to share something useful about today’s coaching (content or process). To understand schools’ implementation needs, participants were asked what future implementation workshops should include. All reflection questions apart from the helpfulness rating were open-ended, qualitative responses. Participants were also asked for the name of the school at which they work, dates of implementation workshop, and what format of coaching they found most helpful, virtual, in-person, hybrid, or no preference.

### 2.7. Analysis

Data analysis in our development evaluation approach was conducted by members of the research team using methods recommended for single case analysis (Yin, 2013, 2015), with the district analyzed as the single case These methods include identification of the research goals (elucidate RPPs development of the implementation supports), guidance by theory and logic of the program being evaluated (implementation strategies for large scale professional development), triangulating multiple sources of information (e.g., notes, agendas, feedback reports, implementation process data), presenting an initial analysis, and collaboratively reviewing analysis to ensure rigor (Yin, 2013).

Our first study goal, which was to focus on describing how the RPP designed a launched a real-world implementation support structure for districtwide professional development, data synthesis, and analysis, was guided by our conceptual models of multi-level implementations strategies and implementation factors (Aarons et al., 2011; Fixsen et al., 2013). To address our research goal of describing the RPP processes used to co-design implementation strategies, the lead authors (research team members) synthesized the information on implementation strategy co-design inputs and outputs and reflected on how these aligned with conceptual model and represented the shared and unique perspectives of the partners (Palinkas & Zatzick, 2019). Coauthors and district partners then reviewed a written summary of the analysis and provided feedback. The feedback helped to enable consensus and refine the analysis. This pragmatic analytic approach prioritizes time efficiency and is designed used for studies with limited resource allocation (Palinkas & Zatzick, 2019).

To address the second research goal of providing examples of the iterative process, a similar process was used. The research team initially reviewed archival documents, agendas, notes, and quantitative implementation process data to select exemplars of the iterative design and refinement process as applied to immediate changes and to longer-term changes to the implementation strategy. District partners and purveyor organization partners reviewed and provided feedback on the selected examples. Of note, in real time, both kinds of sources were used to inform short-term and long-term program development efforts.

## 3. Results

### 3.1. Co-Design of a Professional Development and Training Model

Our first research goal was to describe the RPP processes used to co-design and mount a large-scale professional development program with scalable, sustainable structures and supports. Table 1 depicts an overview of the key elements of the partnered co-design process, highlighting the core contributions of RPP members. The table also aligns elements of the research on implementation strategies. Initial implementation strategies were selected by the RPP, then refined through discussion and feedback, and a workplan for the first-year effort was developed. Each component of the RPP process is detailed below, along with relevant quantitative implementation process data.

#### 3.1.1. Leadership Support and Engagement

The district team saw school administrators (principals and vice principals) as pivotal to the success of the trauma-informed schools’ effort in several ways: their role in the schools, their ability to set the tone and climate at their schools, their communication with district leaders, the need for their buy-in for the effort to be successful, and their ability to allocate staff and resources to the work. As such, in March 2023, before the first cohort training effort was launched, administrators from each school attended an orientation session, co-led by the CSR TISE trainers and district staff. This session included a broad overview of the grant initiative with an emphasis on the TISE activities. These 90 min sessions focused on establishing a rationale for trauma-informed schools. It emphasized the deleterious effects of trauma on key academic outcomes including test scores, exclusionary discipline, and mental health as well as focused on the secondary consequences for educators such as burnout, absenteeism, turnover and secondary traumatic stress. The aim of the session was twofold, to help leaders consider how trauma may be impacting key deliverables and contributing to some of the most common and refractory issues they face in their leadership role, and to gain buy-in to the concept of trauma-informed schools.

#### 3.1.2. Implementation Teams

To move from training to practice change at a large scale across 15 schools, there was recognition across all partners that school-level implementation teams were needed. These teams would need to turnkey materials and practices to school staff and ensure that the training content was connected to day-to-day practices in the schools. These implementation teams would be allotted time to engage in ongoing implementation support with the district. To accomplish this, the district decided to leverage the infrastructure of the district’s newly formed school climate leadership teams (SCLTs). As noted above, the SCLTs were formed to address school climate issues and engage in the state’s school climate improvement initiatives, making their charge well-aligned with the trauma-informed schools’ efforts. In addition, members of these teams also often overlapped with other critical teams, like tier 2 referral and intervention teams and crisis teams. Typically, teams comprised crisis response teachers, counselors, administrators, nurses, and other student support staff.

#### 3.1.3. TISE Training Approach: Asynchronous and In-Person

There was a recognition of the need for engaging training models that could be accessed broadly and have a broad and deep reach within a setting with very limited teacher professional development time. At the same time, the purveyor organization and research team noted that live training may have the benefit of bringing together shared expertise amongst learners, buy in, and the ability to practice skills. To this end, the district opted for a two-pronged approach, asynchronous, web-based interactive training for all school personnel, including non-instructional staff (e.g., student support staff, secretaries, custodial staff, cafeteria staff, afterschool staff) and live training for SCLTs.

The professional development efforts began by delivering the 6 h live TISE training to members of the school climate leadership teams in March 2023. The aim was to equip these individuals with the foundational knowledge that subsequent implementation support and implementation workshops would build upon. Following this, in Fall 2023, the school personnel were asked to take a baseline survey after which they would gain access to the web-based TISE course which could be completed on their own or at a time their school leadership designated. Because of the complexities in rolling this out to 15 schools in a way that accounted for school-by-school burden and needs, and the time needed for all staff to complete this, this timeline for completion was extended from Fall to Spring 2024.

Tracking data showed that across all schools 70% of staff (classroom teachers, custodial, administrative) completed the online course. Five schools achieved TISE-certified status with over 90% of staff completing the asynchronous TISE training course.

#### 3.1.4. Ongoing Coaching or Consultation

All partners also recognized a need for follow-up in the form of coaching or consultation to allow for adaptation and contextualization to improve uptake and sustainability. Since it was not feasible to provide this to frontline staff at all 15 schools, the team opted for monthly follow-up implementation workshop sessions for (SCLT). This included an in-person kickoff event, in September 2023 followed by 90 min virtual workshop sessions from October through May, and in-person site visit to each of the 15 schools, which occurred in January 2024. This hybrid model was deployed to maximize resources and retain some in-person contact, which district partners believed was critical. Overall, there was one in-person group session, four sessions, and an in-person site visit for each of the 15 schools.

In-person launch session. The TISE implementation workshops began the following Fall with a live in-person kick-off event. Following a review of the Spring training, the coaching kick-off centered on three school-wide trauma-informed strategies: community-building circles, classroom mindfulness, and intentional greetings at the school or classroom door. The team also introduced a template for developing action plans related to the use of one or more of these practices. The TISE purveyor organization provided a rationale for each of these strategies and aligned them with trauma-informed principles. For example, classroom circles are intended to foster a sense of belonging, trust and build peer support; mindfulness exercises promote emotional regulation and create a safer school environment, intentional greetings help to establish routine and predictability (SAMHSA, 2014). To enhance a sense of buy-in, research highlighting the positive outcomes associated with these practices was also provided.

Virtual implementation workshop sessions. Subsequent implementation workshops were conducted virtually. During each session, time was allotted for the SCLT to review and revise their implementation action plans for the school- and classroom-wide strategies (i.e., community circles, warm and intentional greetings, mindfulness practices). Additionally, each implementation workshop focused on a trauma-informed principle and an associated trauma-informed skill or strategy. For example, in December the implementation workshop focused on “trust and transparency”. The SCLT members engaged in exercises and reflections intended to help them build trusting relationships with students with an emphasis on those students with whom they have had trouble connecting. The February virtual session introduced data on how child traumatic stress can contribute to burnout and secondary traumatic stress in educators. The session focused on the principle of “peer support”. SCLT participated in exercises intended to help them establish more intentional peer support between staff and cultivate a culture of “collective care”. Virtual coaching in March focused on “cultural responsiveness”. Exercises focused on helping participants identify their own biases and consider strategies to combat these biases and establish a welcoming environment for all students. The final session in May focused on sustainability.

Attendance data reveal that 90 educators (51 unduplicated) attended the four virtual implementation workshops. Of these participants, 75 (83.33%) completed exit tickets. When asked how useful the implementation workshops were on a scale from 1 to 100. These sessions received an average score of 92.12 (SD = 10.08). SCLT were asked to report on the number of trauma-informed strategies being implemented at their school site. All 15 schools reported implementing at least one new trauma-informed practice. The average number of reports by each school was 2.5 (SD = 1.29) and the maximum number of strategies implemented by a school site was 5.

Site visits. Based on experience and observation from district leaders of the initial training and workshop sessions, the district team felt that in-person consultation would be helpful for understanding schools’ immediate support needs and deepening relationships with the SCLTs and administrators at each school. As such, in January 2024, the TISE lead trainer/developer, university partners, and district staff visited each school. The goals were to meet with the SCLTs and learn about their work, understand school needs and priorities, review progress on the TISE asynchronous course completion at the school, review TISE practices and school action plans, and identify immediate support needs (i.e., materials, support requests, modeling and practice). In the majority of schools, site visitors were also taken on a tour of the school.

#### 3.1.5. Practical Support and Resources

To support practice change, schools were given access to practical resources. Some of these were already developed and distributed immediately and others were put into place in response to emergent requests from schools. For instance, the TISE web course came with a set of downloadable PDFs with all the core materials, and the course was available for staff to go back in and review at their convenience. In addition, all the TISE materials were placed into a Google Drive folder available to all school staff. The Drive also included other district-wide MTSS and SEL-related materials aligned with the principles of trauma-informed schools. The district also provided access to the Calm App for all employees to support mindfulness activities and selfcare. Another practical resource was the ability of SCLTs to request on-site support from the district-level implementation team (e.g., demonstration of community circles, support during team meetings for completion of TISE web course, staff “reflection sessions” in which key trauma-informed topics were reviewed and discussed directly with school staff).

#### 3.1.6. Continuous Improvement

Across all components of the implementation support model, continuous improvement (CI) was emphasized. For the SCLTs and administrators supporting TISE course completion and practice use in the schools, CI focused on the development and monitoring of TISE action plans and completion of the TISE course by all school staff. SCLTs also contributed to immediate CI for the RPP that informed the implementation workshop sessions and responsive supports.

Regarding action plans, the university and TISE purveyor organization provided a CI framework (e.g., Plan, Do, Study, Act) and helped the SCLT create an action plan and monitor progress. RPP team members from the district helped to place these activities within the context of district priorities and shared district resources to promote the adoption of the practices. For CI-related TISE completion for individual schools, survey progress was monitored by the university evaluation team as well. Color-coded training and survey completion reports were emailed to each school’s principal and assistant principal on a weekly basis. Completion status was coded as “not started”, “incomplete” or “complete”. The department head from the district central office and the grant’s program director were also carbon-copied on the emails. In the body of the email, the percentage of staff that had completed the survey and online TISE training was included and highlighted. Schools were incentivized to reach 90% online TISE completion with a banner they could hang in their entryway pronouncing the school a TISE-certified school.

CI was also used to make short-term and long-term changes to project implementation supports. Feedback from administrators and SCLTs to the RPP members was provided in several ways, including pre and post surveys, site visits, verbal feedback and informal exchanges, and exit tickets in which SCLT members rated each training and workshop session, provided updates on their team’s progress, and made suggestions for future sessions and support needs. Once we received feedback, regardless of the source, we analyzed and interpreted the feedback data with RPP team members and then jointly planned an appropriate response.

An example of changes made based on CI data was learning that schools were interested in “turnkey” materials and supports via site visits and exit tickets. Accordingly, the support team was able to respond immediately with materials and targeted modeling of skills. For the next cohort of schools, this feedback allowed the RPP to ensure that the materials and practice needed to enhance SCLTs’ abilities to turnkey the TISE skills to staff were addressed from the outset. Additionally, conversations from implementation workshops revealed that some schools had higher TISE course completion rates when staff were offered dedicated time to complete modules during 30 min morning staff meetings. These data are being used by the RPP team to inform the asynchronous roll-out for the next cohort of schools to include dedicated time for staff to complete TISE.

#### 3.1.7. Relationships

Underlying all the activities was a concerted effort by the RPP team to build and maintain relationships. This need to tend to relationships and maintain strong connectedness is an implementation strategy that was continually attended to and elevated by the district members of the RPP. For instance, the district was clear that relationships with and responsiveness to school principals and assistant principals were critical to obtaining critical buy-in and supporting uptake of the training. As such, school administrators were engaged early in the process and communicated regularly through formal and informal channels. These administrators were also encouraged to provide feedback and share their needs and concerns. The district also advocated for in-person components to be built into the implementation workshop series (site visits, in-person kick off session) to establish connectedness across schools and with the RPP team. The site visits were excellent vehicles to build a sense of collaboration, mutual trust, and responsiveness to school needs because the TISE team solicited concerns about the PD effort from administrators, and provided immediate follow-up. Specifically, during the January site visits, school administrators from five schools indicated that they were having challenges turnkeying the TISE practices to their educators. Though live training from the TISE team for non-administrative educators was not initially a component of our professional development model, the TISE team responded to this request by building out a new structure called “TISE reflection sessions.” TISE reflection sessions were in-person mini-trainings focused on specific trauma-informed skills for educators. Sessions included direct instruction, discussion, practices, and planning. Reflection sessions were provided within three weeks of the site visits.

In addition, for the SCLTs, the use of exit tickets and informal emails communicated an openness to feedback. Timely responsiveness to that feedback was provided with the intention of strengthening the relationships and enhancing mutual trust and support. This responsivity was not limited to TISE implementation. For instance, SCLTs could contact the district team for support around any need (i.e., crisis response, consultation, professional development needs, connections to other community organizations). University and team members provided support through resources and occasional school visits and meetings. The district team also regularly connected with their school-level colleagues informally through in-person visits and tended to interpersonal relationships through participation in celebrations, community events, and informal and formal recognition.

### 3.2. Case Examples of Feedback Loops for Iterative Change

The second goal of this study was to share specific case examples of data-driven process improvement efforts that were used in real time. Case examples were selected by the research team, and reviewed by the entire RPP, with two primary criteria: (1) that they illustrate an immediate, short-term improvement process and longer-term improvement issue, and (2) that they each represented a different component of the implementation models (e.g., training, coaching strategies, implementation teams, uptake). All short-term and long-term feedback loops followed the same workflow to inform CI efforts—receipt of feedback data ⟶ Analysis of feedback data with RPP team members ⟶ Collaborative interpretation of the feedback ⟶ Joint planning of appropriate response by the research team, school district team, and the purveyor organization team. Regardless of feedback source (e.g., verbal, written, formal, informal, routine, spontaneous, qualitative or quantitative), CI decisions were made following this same process.

To illustrate the RPP’s iterative program development and user-centered design processes, we will share two case examples of feedback loops, an immediate feedback loop and a long-term feedback loop. The first case example will focus on the processes used to collect feedback for immediate implementation—the exit ticket. The second case example will highlight a data source used for long-term program development efforts—coaching attendance patterns.

#### 3.2.1. Case Examples for Immediate Feedback—Exit Ticket Feedback Loop

This section will first describe the exit ticket feedback loop process and then provide specific examples of each step from the RPP.

##### Overview of Process

**Figure 1:**
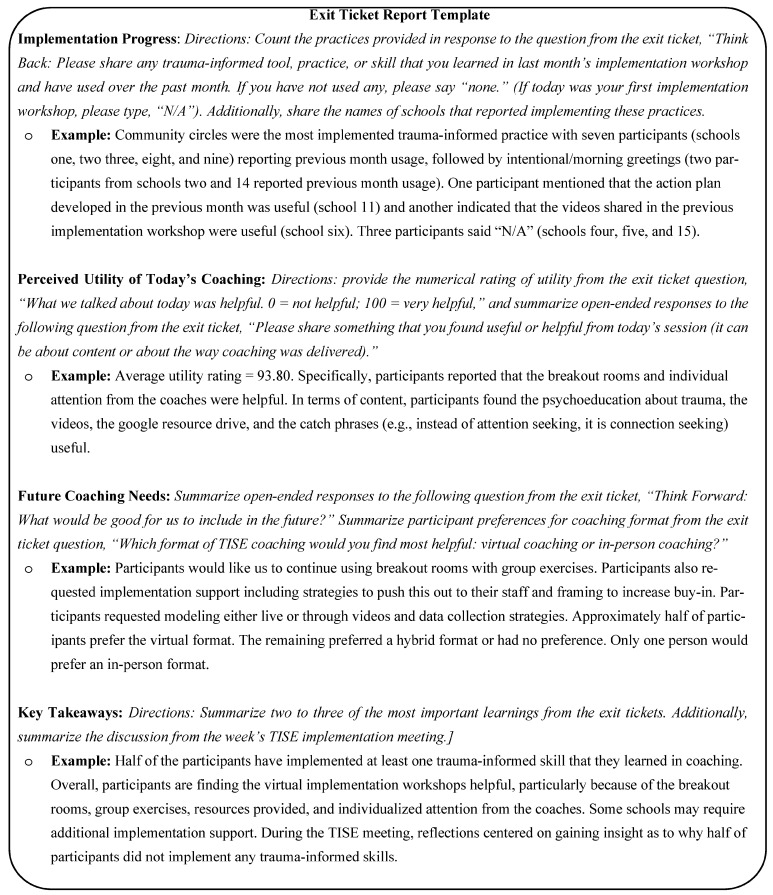

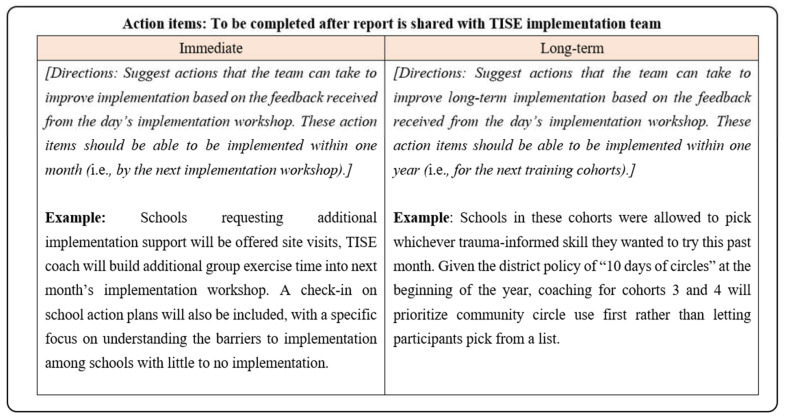
Exit ticket report template with de-identified examples.

Exit tickets were completed at the end of each implementation workshop and training. Once collected, a research team member emailed the results to TISE team members immediately following the workshop session. The research team member also summarized the results and shared this summary with the implementation team at the next weekly team meeting using the “Exit Ticket Report” format (see Figure 1 for the Exit Ticket Report template). Results were typically shared verbally with the team. The TISE implementation team then discussed the results and proposed modifications to the TISE implementation support strategies. District personnel on the TISE team provided context to the results with information about each school’s needs, resources, and capacity, and broader district policies and initiatives. Researchers on the TISE team facilitated the interpretation of the exit ticket report results and propose next steps best practices in the literature. Researchers were also able to clarify any questions about the data collected. Representatives from the purveyor organization used the input from district personnel and researchers to make changes to the implementation workshop curriculum. Since the purveyor organization led the implementation workshops, they were also able to provide additional insight to the exit ticket results based on their real-time observations.

##### Specific Feedback Loop

To illustrate this process, we will describe the exit ticket feedback loop from December of 2023. The virtual implementation workshop with SCLTs took place on 7 December 2023 for 90 min. This workshop was led by a research team member with the TISE trainer and supported by a district team member. At the conclusion of the workshop, 15 of the 27 attendees completed the exit ticket. Exit ticket results were emailed to all TISE implementation team members and summarized by a research team member who then presented the summary to the TISE implementation team two business days later at the implemented team weekly meeting. Team members present included one school district project director, one project clinician, three research team members, and three staff from the purveyor organization. The primary findings from the exit tickets were that the school leaders who had attempted to implement a trauma-informed practice overwhelmingly chose to implement community circles, school leaders found the implementation workshops very useful particularly because of the hands-on exercises and breakout rooms, and in the future, they would like additional implementation supports.

School district leadership present at the meeting were able to contextualize the strong community circle implementation in the broader landscape of district policy by sharing that the district put out a “10 days of circles” challenge to the schools. Each school was encouraged to implement community-building circles for 10 consecutive days. Understanding this context was invaluable to interpreting the results of the exit ticket and designing short-term and long-term action items. If district personnel would not have been members of the implementation team, that information may have been lost, leading to inaccurate interpretations of the results and unhelpful adjustments to the short-term and long-term implementation strategy.

To address the need for additional implementation supports, materials were developed to facilitate the SCLTs ability to “turnkey” lessons and exercises from the implementation workshops. These materials included brief PowerPoint decks that the SCLT members could present during school staff meetings to deepen their colleagues’ understanding of a particular trauma-informed principle or skill. These decks were accompanied by modified versions of the handouts and exercises used during the implementation workshops. For example, one slide deck focused on “trust and transparency” and consisted of five slides that emphasized the concept of protective factors with an emphasis on trusted adults. The associated handout was a modified version of the building relationships exercises conducted in the December implementation workshop.

#### 3.2.2. Case Study for Long-Term Feedback—Attendance Data from Implementation Workshops

This section will first describe the attendance data feedback loop process and then provide specific examples of each step from the RPP.

##### Overview of Process

Attendance was collected using a Google Form at the beginning of each implementation workshop and training. At the end of the year, once all implementation workshops had been completed, the TISE implementation team discussed the attendance results and proposed strategies to improve SCLT attendance during the next school year. District personnel on the TISE team provided context to attendance with information about schools’ scheduling needs, turnover, and broader district policies, such as who provides coverage for administrators when they attend professional development. Researchers on the TISE team facilitated the interpretation of the attendance results, proposed next steps best practices in the literature, and clarified any questions about data collection. Representatives from the purveyor organization used the input from district personnel and researchers to make changes to the professional development program. Attendance data were also used for immediate improvement following each implementation workshop (e.g., sending reminders to school administrators to attend the next workshop), but the focus of this case study will be on the long-term feedback loop.

##### Specific Feedback Loop

Upon the conclusion of the 2023–2024 school year, a research team member consolidated the attendance data from all four implementation workshops. Attendance data were visualized using a pivot table to better identify patterns (see Table 2 for an example). Across the academic year, all 15 schools attended at least one implementation workshop with the average school attending 2.31 workshops (SD = 1.08). A total of 50 unduplicated SCLT members attended the four implementation workshops. The average attendance at each workshop was 18 (SD = 6.93). The most highly attended workshop was the first on 7 December 2023, with 27 participants. The workshop with the lowest attendance was the final workshop on 10 May 2024, with eight participants. Two participants attended all four sessions, and 32 participants attended only one session (M = 1.61 sessions, SD = 0.85).

Based on these patterns of attendance, the research team was able to conclude that, though each implementation workshop was well attended, each participant’s attendance was highly inconsistent. While the goal of these implementation workshops was that all SCLT members would attend each workshop, it appeared that instead, in most schools, SCLT members were taking turns attending. This finding provided valuable information about how SCLTs were conceptualizing implementation and operationalizing their roles in the process and highlighted an area for strategic adjustment. TISE implementation team members from the school district offered two possible interpretations of this finding, (1) messaging to SCLTs about attendance expectations was unclear, so to minimize disruptions to the schedule and need for coverage, SCLTs selected 1–2 team members to attend each implementation workshop and communicate back their learnings, or (2) as a newly formed team, SCLTs were still developing and thus somewhat amorphous, with evolving membership, goals, and procedures. These results surfaced important insights related to internal coherence and readiness for implementation and the real-world challenges that school districts can face in launching new programs at a large scale. Further, these results demonstrated the necessity of CI in identifying and responding to contextual dynamics within each school.

In response, the TISE implementation team developed targeted adjustments to the professional development program for the second year of the project. The year two training plan would integrate more work around building a cohesive team and team consistency and include explicit and continuous evaluation of readiness to implement trauma-informed principles. Specifically, the plan included incorporating team-building content at the beginning of the year kick-off event, acknowledging the “multiple hats” worn by many team members, providing trauma-informed leadership training to all administrators emphasizing team-based approaches to trauma-informed schools, and highlighting the expectations of consistent team membership and participation in all communication materials regarding implementation workshops. To evaluate readiness for implementation, questions related to a readiness-for-change would be included in the exit tickets. Finally, the TISE implementation team also decided that a mid-year attendance check should be included in year two, in addition to the end-of-year analysis, to allow for short-term feedback loops to utilize attendance data as well.

This feedback loop and its resultant adaptations to the professional development program illustrate the utility of the developmental evaluation approach to co-creating school-based professional development. This approach allowed the RPP team members to track implementation progress while simultaneously addressing real-world implementation challenges in a responsive and collaborative manner.

## 4. Discussion

This mission of K-12 schools in the United States is to “promote student achievement and preparation for global competitiveness by fostering educational excellence and ensuring equal access” (Department of Education, 2024). However, studies clearly demonstrate that exposure to traumatic and adverse experiences in youth can hinder academic achievement, impede students’ social–emotional development, and increase their likelihood of mental health challenges (Perfect et al., 2016). There is also growing evidence that the COVID-19 pandemic increased exposure to potentially traumatic experiences for children and adolescents as moderate to severe pandemic-related stress in caregivers was correlated with a 108-141% increase in ACE exposure (Thierry et al., 2023). While teachers are well-positioned to support students as part of trauma-informed school initiatives, they lack certainty in their ability to do so effectively (Alistic, 2012). Isolated professional development training has been shown to improve attitudes and knowledge about student mental health needs; however, this “train and hope” approach it is less effective in driving sustainable practice and behavior change (Adrian et al., 2018). Moreover, existing coaching and consultation models have shown promise in supporting practice use and quality (e.g., Ennis et al., 2020; Erchul, 2023; Pas et al., 2023). However, many of these have been used in the context of research studies (Lyon et al., 2024), and need to be tailored to match the needs, resources, and structures of the school districts as part of district-led efforts.

The current study contributes to the implementation of trauma-informed school literature by describing the co-design process for implementation supports and structures for a schoolwide, trauma-informed professional program. RPP and user-centered design have been shown to improve fitness, implementation and sustainability of practices across a variety of settings (e.g., Coburn & Penuel, 2016; Haines et al., 2021). This developmental evaluation of a co-designed trauma-informed professional development strategy for educators within a large urban school district aligns with these findings. Specifically, our trauma-informed professional development strategy emphasized school district leadership support and engagement; school-level implementation teams and received ongoing implementation support for these teams in the form of implementation workshops; in-person engagement events, leadership training, and on-site technical support; a virtual, asynchronous, trauma-informed curriculum to distribute to all staff; tangible training materials on trauma-informed schools; continuous improvement mechanisms built into the structure of the PD; and a heavy emphasis on relationships. Critically, while prior research has focused primarily on small-scale pilot efforts or externally driven initiatives, this study highlights how district-led, co-designed professional development programs can be responsive to local context and implemented at scale. This approach is consistent with the teacher professional development literature which calls for practitioner ownership and leadership of professional development efforts (Postholm, 2018). Our results have added an understanding of how outside resources including purveyor organizations and researchers can contribute to professional development in collaboration with school districts by providing content area expertise and regularly scheduled, responsive implementation support.

Our results additionally align with the trauma-informed school training literature, which emphasizes three dimensions of an effective approach: teacher professional development, practice change, and organizational change (Avery et al., 2021). Teacher professional development was provided directly by the purveyor organization in the form of the TISE asynchronous online course. PD was also provided to teachers by SCLT members. Trauma-informed practice changes included the implementation of community circles, positive affirmation boards, and intentional greetings. Organizational-level changes included leadership support and engagement, ongoing implementation support, and continuous improvement mechanisms. Our study also addressed a key gap in the trauma-informed schools and implementation literature around the role of relationship-building as an implementation strategy.

Relationships are often listed as an implementation strategy and are routinely discussed as a key element of program development, implementation, and coaching (e.g., Powell et al., 2011). Additionally, many commonly cited implementation strategies have been identified as being highly relational, characterized by frequent interactions, shared power, mutual growth, accountability, and potential vulnerability (Bartley et al., 2022). However, there are few studies that highlight the specific ways relationships are cultivated as an implementation strategy. Relationships were seen by the district team as foundational in terms of trust and connectedness, learning together, and authenticity. It was also important to the district that the implemented supports were responsive to school leaders and staff. In the current study, this was emphasized with school personnel, but relationships and trust have also been found to be a key feature of strong RPPs (Henrick et al., 2017). Metz et al. (2022) have put forth a conceptual model of trusting relationships in implementation work which highlights the role of both relational strategies (i.e., authenticity, vulnerability, bi-directional communication, co-learning, empathy-driven interactions) and technical strategies (i.e., frequent interactions, responsiveness, demonstration of expertise, quick wins) as key drivers of positive, safe, and trusting relationships. This, in turn, impacts motivation, commitment, and implementation outcomes.

Building on this strong foundation of collaboration and partnership, the current study also represents an application of dynamic, iterative partnership methods and research methods that can be deployed in other settings. As noted above, we are building on the literature related to trauma-focused professional development as well as delineating strategies that can be used for user-centered design and co-design in schools that are launching their professional development and implementation efforts. We were also able to apply rapid qualitative methods in ways which led to immediate actionable steps within our partnership and for our schools. The use of qualitative methods and case study analysis are useful for building knowledge in a complex field and can inform the development of strategies that can be tested using other study designs.

There are important limitations to the study. While our study focused on the educator-facing structures needed (i.e., professional development and accompanying implementation support) to build capacity for a trauma-informed approach in this school district, we recognize the importance of engaging student voices in trauma-informed school efforts. Other branches of this grant-funded work include an emphasis on student voice, choice and empowerment including student-led wellness clubs. Future phases of this partnership have additional planned mechanisms for gathering student perspectives and integrating them into the trauma-informed initiatives and practices we are developing. These planned mechanisms include the use of student-level school climate data, universal mental health screening data, and focus groups.

As in any single case study, there are core issues related to generalizability and representativeness. The school district in the current study was supported by grant funding, which allowed them the funds to obtain training and provide ongoing support. Not all districts will have such resources. Also, the district itself is a relatively large, diverse, and urban district and may have unique characteristics that may not generalize to non-urban settings. In addition, while our research team is investigating implementation outcomes from this effort, the lack of a comparative design makes it impossible to draw conclusions about the effectiveness of the implementation strategies we deployed.

Even with these caveats, there are several lessons that are generalizable to other settings. Many districts use grant funds and existing partnerships to launch new initiatives and build infrastructure for school mental health services, SEL programs, MTSS, and other similar efforts focused on student’s well-being and academic success. The current study offers specific strategies, considerations, and processes that can inform the district’s efforts to determine the scale and scope of their implementation efforts, select strategies, and tailor them for their own contexts. In addition, the way the district team-centered relationships in the current effort and leveraged the expertise available to them in the partnership itself is also informative. It is also critical to note that the district knew the needs of their setting in a way the research team and purveyor organization could never have. This local expertise is critical to the implementation and sustainment of programs.

## 5. Conclusions

Creating trauma-informed schools remains an educational and public health priority. The co-developed TISE professional development program for schools and accompanying implementation supports represent a promising model for districtwide implementation of trauma-informed principles and training for educators. The partnered approach to development, implementation, and continuous improvement, particularly the intentional approach to building relationships, was a key driver of the program’s promising start. Importantly, substantive systemic change requires time. Our project is a multi-year effort, and this case study presented data from the first year of implementation. It is too often the case that systemic change efforts within schools fall short of the mark because they are not viewed as permanent by school staff, but rather as temporary initiatives (e.g., the “flavor of the month”) put in place because there was an influx of temporary grant funding or because the approach is in vogue but will fall out of favor to be replaced with another shortly (Adelman & Taylor, 2007). Creating a multi-year implementation strategy for the TISE PD program was necessary in combatting this tendency and allowing for continuous improvement and tailoring. Further, our PD specifically included a system and practice change lens. This is important because if systemic change is an afterthought in school improvement efforts, little or no sustained improvement results (Adelman & Taylor, 2007). To our knowledge, researchers have not yet explored how to co-create multilevel, trauma-informed professional development programs and scale them to the school district level. Our RPP components and processes along with the concrete examples described in the case studies represent an effort to address this gap, and our results may help inform others engaged in trauma-informed school research.

## Figures and Tables

**Table 1:** Overview of the key components of the partnered co-design process.

	RPP Co-Creation Process			
Implementation strategies (from the literature)	Research Team Expertise (e.g., Implementation factors and drivers)	Purveyor Organization Technical Assistance	District Expertise (e.g., needs, priorities, structures, capacity, prior experience)	Co-designed implementation strategies
Provide Leadership support and engagement	Organizational climate for implementation Practical and transformational leadership	Prior experience with schools who had communicated mission, visions, and alignment	Recognition of the important role and expertise of school principals Need for high-level support of school-level implementation teams Connection to district leaders	Principal engagement sessions Direct principals’ communication with district, CSR, and university staff Communication with official district memos
Create Implementation Teams	Multidisciplinary and multi-level Team effectiveness Organizational climate for implementation	Experience with coaching and leadership support	Staff on multiple teams with multiple roles Time constraints for team meetings Need to leverage existing infrastructure	Leverage newly formed school climate leadership team
Identify trauma-informed schools training curriculum	Penetration and reach of practices for a whole school approach Fitness, relevance, ease of use, evidence-based content	Subject matter expertise Flexible options	Limited professional development time Prioritize accessibility and flexibility Desire to reach all school personnel in all schools Building expertise	Asynchronous, self-paced, web-based training with interactive elements Live, interactive training for school climate leadership teams
Provide ongoing coaching or consultation	Practice, coaching, and feedback Shared peer-to-peer learning Accountability	Virtual support informed by continuous feedback	Lack of resources to coach all frontline staff Direct observation classroom practice not feasible	Implementation workshop model Virtual In-person kick off Individual site visits in person
Deliver practical resources	Utility Ease of use Trailable and adaptable	TISE worksheets Other implementation materials (e.g., demonstration videos) Google Drive of resources	“Turnkey” materials (scripts, videos) Easy access	Google Drive/Padlet Access TISE web course (including review videos and PDFs) Live modeling upon request Calm App
**Build and maintain relationships**	Frequent, informal communication Knowledge of effective workplace relationships (i.e., consistent positive communication must outweigh demands)	Trauma-informed principles of communication (trust, safety, transparency, voice, choice, and empowerment)	Knowledge of communication pathways within the district Prior relationships Knowledge and prior experience	Creation of direct and responsive communication channels (e.g., text, personal cell phone calls, in-person drop ins) Responsive model to ensure timely support is provided
**Engage in continuous improvement**	Feedback loops between frontline providers and leadership	Responsivity to feedback Flexible support model	Knowledge of CI in education Feedback in low-burden methods for CI	Staff survey completion TISE completion Exit tickets Workshop attendance

**Table 2:** Sample attendance pattern across implementation workshops.

	7 December 2023 or 8 December 2023 ^a^	23 February 2024	22 March 2024	10 May 2024	Total Number of Sessions Each Participant Attended
School 1					
Participant 1	1				1
Participant 2	1				1
Participant 3	1		1		2
Participant 4	1				1
School 2					
Participant 1	1	1			2
Participant 2			1	1	2
Participant 3				1	1
Participant 4	1				1
Participant 5	1				1
Participant 6	1		1	1	3
Participant 7	1				1

^a^. December’s implementation workshop was offered twice to accommodate district scheduling needs. SCLT members attended the session that fit their schedule.

## Data Availability

The raw data supporting the conclusions of this article will be made available by the authors on request.
